# Effect of the Interplay between Trauma Severity and Trait Neuroticism on Posttraumatic Stress Disorder Symptoms among Adolescents Exposed to a Pipeline Explosion

**DOI:** 10.1371/journal.pone.0120493

**Published:** 2015-03-20

**Authors:** Wei Guo, Jiao-Mei Xue, Di Shao, Zhou-Ting Long, Feng-Lin Cao

**Affiliations:** School of Nursing, Shandong University, Jinan, Shandong Province, People’s Republic of China; Univ of Toledo, UNITED STATES

## Abstract

**Background:**

While numerous studies have explored relevant factors of posttraumatic stress disorder (PTSD) symptoms, there have been few joint investigations of trauma severity and trait neuroticism on the development of PTSD symptoms. This study aims to assess the involvement and interrelationship of trauma severity and neuroticism in the expression of PTSD symptoms among adolescents exposed to an accidental explosion.

**Methods:**

Six hundred and sixty-two adolescents were recruited from a junior middle school closest to the 2013 pipeline explosion site in China and were assessed using the Explosion Exposure Questionnaire, the NEO Five Factor Inventory-Neuroticism Subscale (FFI-N), and the PTSD Checklist-Civilian (PCL-C). A battery of hierarchical multiple regression analyses and two-way ANOVAs were performed to examine the effect of trauma severity and trait neuroticism on adolescent PTSD symptoms.

**Results:**

Eighty-seven adolescents (13.1%) showed PTSD symptoms after the pipeline explosion. Correlation analysis showed that all the factors of explosion exposure and trait neuroticism were positively associated with adolescent PTSD symptoms. Being male and younger was linked to lower risk for PTSD symptoms. The regression models identified explosion exposure and neuroticism as independent risk factors for PTSD symptoms, and the interactions between trait neuroticism and trauma exposure (personal casualty, degree of influence, total traumatic severity) were related to PTSD symptoms.

**Conclusions:**

The results highlight the role of trauma exposure and trait neuroticism as risk factors for PTSD symptoms. Therefore, the combination of these two factors should be investigated in clinical settings due to an augmented risk for more severe PTSD symptoms.

## Introduction

In the past two decades, destructive events have caused millions of deaths worldwide. Sudden unexpected traumatic accidents, such as hurricanes, tsunamis, earthquakes, mudslides, traffic accidents, airplane crashes, and explosions, have led to a broad range of serious and long-lasting negative physical and psychological consequences for both children and adult survivors. These include internalizing and externalizing psychopathology such as anxiety, depression, posttraumatic stress disorder (PTSD), borderline personality disorder, substance abuse, antisocial behaviors, suicidality, somatic complaints, nightmares, and overall reduced functioning [1–5].

Posttraumatic stress disorder is usually considered the most prevalent psychopathology among juvenile populations exposed to deadly disasters [6–8]. PTSD is a series of chronic emotional responses to traumatic events or situations involving a high level of environmental stress, with symptom clusters of re-experiencing, avoidance, and hyper-arousal [9,10]. It has been documented that the prevalence of PTSD is 3.7%–93.0% for adolescents exposed to war [11,12], 25%–30% for survivors following road traffic crashes [13], 19%–27% following cardiac arrest [14], and 0%–95% following natural disasters [15–19]. This large variability in prevalence rates is likely attributable to several variables, such as the age of individuals, nature of the disaster, proximity to the trauma, methodological variations relating to measures used, time elapsed since the exposure, and study design [14,20,21]. Accruing empirical evidence has shown that PTSD is associated with adverse health behaviors (e.g., alcohol and substance use, smoking, physical inactivity, HIV risk behaviors, suicide) that increase the risk of morbidity and mortality [22–26].

It has been suggested that individuals exposed to technological accidents would show greater emotional responses than those who experienced natural disasters [27]. In November 2013, a terrible pipeline explosion occurred in a city located in east China. Schools, factories, and residential areas around the bomb site were severely influenced. Despite the increasing evidence of a high prevalence of PTSD symptoms among children and adolescents after natural disasters, there are no previous studies on the prevalence of PTSD symptoms in a large sample of school-aged adolescents exposed to explosion. Therefore, it is necessary to gain an understanding of the prevalence rates and risk factors related to the development of PTSD symptoms among this special population, to provide the most timely and effective interventions.

As mentioned previously, a considerable body of research has examined prevalence rates of PTSD, as well as relevant risk factors, among victims of natural disasters and war-related bombing [17,28–33]. The construct of stressors is fundamental to the field of developmental psychopathology [34]. At the theoretical level, the majority of common models of child/adolescent psychopathology emphasize the potential impact of environmental stressors in the etiology and course of both internalizing and externalizing disorders in youth [35–38]. Studies have found empirical support for these theories. Severity of exposure to disasters (i.e., injury, witness of injury or death, loss of house or property, injury or death of family members and/or relatives, perceived threat) was the most evident risk factor for adolescent mental health [39–42]. Additionally, history of multiple trauma types distinctly increased the likelihood of subsequent PTSD symptomatology [43], suggesting that greater exposure may result in increased levels of PTSD symptoms.

Even though stress is a major risk factor for PTSD symptoms, considerable heterogeneity exists in outcomes after stress, and a large proportion of individuals exposed to early-life stresses will not exhibit psychopathological symptoms as adults [44,45]. Therefore, the adverse effects of stress may surface most readily in individuals at increased risk for PTSD. Previous research has indicated that level of neuroticism contributes to vulnerability to PTSD symptoms [46,47]. According to the biopsychosocial model of psychopathology, mental disorders result from the interaction of biologically based dispositional vulnerabilities and traumatic experiences. Several studies support this model. Previous lines of evidence have suggested that neuroticism interacts with stressful life events to trigger new episodes of recurrent depression among populations of all ages [39,48]. Martin-Blanco et al. (2014) concluded that the interaction between temperamental characteristics and early-life emotional abuse has an influence not only on the development, but also on the severity, of borderline personality disorder [49]. Therefore, we hypothesized that trauma exposure could particularly increase the risk of PTSD symptoms in individuals with high levels of neuroticism.

It is worth noting that few previous studies have jointly investigated both personality characteristics and trauma severity in relation to PTSD risk factors. Accordingly, the current study will focus on prevalence estimates among adolescents between 10 and 16 years of age, and the involvement and interrelationship of trauma severity and trait neuroticism in the development of PTSD symptoms following explosion-related trauma. Specifically, we hypothesized that: (1) each aspect of trauma severity (i.e., personal casualty, property loss, degree of influence, total traumatic severity) would be positively correlated with PTSD symptoms; (2) trait neuroticism would be positively associated with PTSD symptoms; and (3) trauma severity would interact with trait neuroticism, leading to PTSD symptoms in adolescents, and the association between trauma severity and PTSD symptoms would be most pronounced in individuals with high levels of neuroticism.

## Materials and Methods

### Design

This study was a cross-sectional survey of adolescents exposed to an accidental explosion in China. Each participant was administered a short questionnaire to collect demographic data and a set of self-reported questionnaires one month after the accidental explosion.

### Participants

Initially, a sample of 788 participants was recruited from a public junior middle school (7^th^ and 8^th^ grade) located in east China near the 2013 explosion. This middle school was located closest to the bomb site and some buildings were destroyed and students were moved to another school. The only exclusion criterion for the study was refusal to participate, yielding a response rate of 85%. Finally, data for 662 respondents were complete and suitable for analysis. The demographic characteristics of participants are described in the Results section.

### Ethics statement

Ethical approval for the study was obtained from the Research Ethics Committee of Shandong University School of Nursing, and permission to carry out the study was granted by the principal of the school and classroom advisers for each class. Written informed consent was obtained from participants and their parents and they were guaranteed anonymity, confidentiality, and the right to withdraw from the study at any time. Research assistants explained the purpose of the study to all students in their classes and provided them with standardized instructions orally to ensure successful completion of the questionnaires.

### Measures

#### Demographic data

Socio-demographic information collected for this study included age, gender, and current grade.

#### Severity of trauma exposure

Severity of trauma exposure was assessed with the Explosion Exposure Questionnaire, which was consulted and adapted from scales used in prior studies focusing on natural disasters [50]. Six items covered three aspects of exposure to the explosion incident: (a) Personal casualty (3 items): injury or death of any family member or relative due to the accident (none, injury, death or loss); injury or death of any acquaintance (besides relatives) due to the accident (none, injury, death or loss); witnessing the injury or death of someone due to the accident (no, yes), (b) Property loss (2 items): extent of the impact of the explosion on their house (none, slight, moderate, severe); extent of the impact of the explosion on their property (none, slight, moderate, severe), (c) Degree of influence (1 item): degree of the impact of the incident on the respondent (none, slight, moderate, severe).

#### PTSD symptoms

PTSD symptoms were assessed using the PTSD Check List-Civilian Version (PCL-C), a self-rated instrument measuring PTSD symptoms, with 17 items corresponding to the symptoms emphasized in the DSM-IV: PTSD Criteria B (re-experiencing), C (avoidance/ numbing), and D (hyper-arousal) [10]. The response options for each item on the PCL-C were rated on a five-point Likert scale from 1 (*“not at all”*) to 5 (*“extremely”*) based on the degree to which a respondent had been troubled by a specific symptom during the past month. Total scores are calculated as the mean response across all items, ranging from 17 to 85, with a higher total score representing greater PTSD symptoms. The recommended cutoff point for screening PTSD due to sudden traumatic accidents is set at 38 [51]. The Chinese version has been shown to have acceptable psychometric properties [52].

#### Trait neuroticism

The NEO Five- Factor Inventory (NEO-FFI) [53], is a shortened version of the NEO PI-R, which contains scales assessing Neuroticism, Extraversion, Openness, Agreeableness, and Conscientiousness, with 12 items per dimension. The NEO-FFI consists of 60 five-point Likert-scaled items, with response categories from 1 (*“strong approval”*) to 5 (*“strong disapproval*”). In the current study, neuroticism was assessed by the Neuroticism subscale. Higher total scores are indicative of greater levels of neuroticism. The NEO-FFI has been shown to have satisfactory construct validity and good internal consistency in general population samples [54].

### Statistical analysis

IBM SPSS Version 21.0 was used for statistical analysis. Data were double entered after careful review for completeness and obvious errors. Descriptive statistics including frequencies, means, and standard deviations (*SD*) of the demographic variables were computed. We utilized the mean of nearby points to substitute for missing data. Interrelations between the main psychosocial variables were tested using Pearson correlation analyses. The association between demographic variables (including age and gender), trauma severity, trait neuroticism, and PTSD symptoms was examined using a set of hierarchical multiple regression analyses. In all analyses, PTSD symptoms were regarded as outcome variables. All independent variables were centered on their respective means in order to reduce the problem of multi-collinearity between main effects and the interaction term, as well as to enhance the predictive power of β weights for interaction terms. Finally, a series of two-way ANOVAs were performed to further illustrate the significant interactions. All tests were two-tailed with *P* <. 05 representing statistical significance.

## Results

### Sample demographics and trauma characteristics

The final sample was composed of 622 trauma-exposed adolescents. Demographic characteristics and trauma characteristics of the sample are summarized in Table 1. Notably, the majority of adolescents (78.5%) witnessed the injury or death of someone due to the accident. According to scores on the PCL-C, 87 adolescents (13.1%) showed PTSD symptoms.

**Table 1:** Demographic and descriptive data of the study population (*N* = 662).

		*N* (%)	*M (SD)*
***Demographic characteristics***			
Age (years)			13.05(0.83)
Gender	Male	365 (55.1)	
	Female	297 (44.9)	
Grade	7th	371 (56.0)	
	8th	291 (44.0)	
Type of trauma	Injury/death of family member	17 (2.6)	
	Injury/death of friends	115 (17.4)	
	Witnessed injury/death	520 (78.5)	
	House damage	68 (10.7)	
	Property loss	47 (7.1)	

### Trauma severity factors and trait neuroticism associated with PTSD symptoms

Table 2 shows the means, standard deviations, observed ranges, and results of bivariate correlations of the study variables. As expected, all factors related to explosion exposure (i.e., personal casualty, property loss, degree of influence, total trauma severity) and trait neuroticism were positively related to adolescent PTSD symptoms. Moreover, girls suffered more severe PTSD symptoms than boys did. Additionally, younger age was associated with lower risk for PTSD symptoms.

**Table 2:** Pearson correlations, means, standard deviations (SD), and observed ranges of study variables (N = 662).

	1	2	3	4	5	6	7	8	*M*	*SD*	Observed range
1 Age									13.05	0.83	10–16
2 Gender ^a^	−.065										
3 Personal casualty	.066	.009							4.07	0.79	3–8
4 Property loss	−.022	.009	.155**						2.25	0.75	2–6
5 Degree of influence	.122**	.079*	.214**	.102**					1.99	0.90	1–4
6 Total trauma severity	.079*	.043	.797**	.263**	.593**				1.30	0.91	0–5
7 Neuroticism	.099*	.079*	.158**	.027	.236**	.219**			27.81	8.76	12–60
8 PTSD symptoms	.148**	.142**	.301**	.118**	.602**	.492**	.419**		26.72	11.84	17–85

Note. PTSD = Posttraumatic stress disorder;

^a^0 = female, 1 = male;

*P < 0.05

**P < 0.01.

### Interactive effect of trauma severity and trait neuroticism on PTSD symptoms

To examine the effect of trauma severity and trait neuroticism on PTSD symptoms, we performed a battery of hierarchical regression analyses. Table 3 shows the results of the subsequent regression models considering PCL-C total score as the dependent variable, age and gender as control variables, and one of the four indicators of trauma severity (e.g., personal casualty), trait neuroticism, and the interaction term involving trauma severity factors and trait neuroticism as independent variables. After controlling the effect of age and gender, each indicator of trauma severity (i.e., personal casualty, property loss, degree of influence, total trauma severity) were significantly positively correlated with adolescent PTSD symptoms. Of note, interactions between trait neuroticism and all indicators of trauma severity (except property loss) were significantly related to PTSD symptoms.

**Table 3:** Trauma severity and trait neuroticism as predictors of posttraumatic stress symptoms among adolescents exposed to an accidental explosion.

Variables	β	*P*	Δ*R* ^2^
Step 1: Control variables			.052
Age	.18	.000	
Gender^a^	.15	.000	
Step 2: Independent variables			.196
Personal casualty	.23	.000	
Neuroticism	.35	.000	
Step 3: Interaction			.012
Personal casualty × neuroticism	.11	.001	
Step 1: Control variables			.052
Age	.18	.000	
Gender^a^	.15	.000	
Step 2: Independent variables			.161
Property loss	.13	.000	
Neuroticism	.38	.000	
Step 3: Interaction			.000
Property loss × neuroticism	.00	.884	
Step 1: Control variables			.052
Age	.18	.000	
Gender^a^	.15	.000	
Step 2: Independent variables			.422
Degree of influence	.54	.000	
Neuroticism	.27	.000	
Step 3: Interaction			.008
Degree of influence × neuroticism	.09	.002	
Step 1: Control variables			.052
Age	.18	.000	
Gender^a^	.15	.000	
Step 2: Independent variables			.329
Total trauma severity	.44	.000	
Neuroticism	.30	.000	
Step 3: Interaction			.011
Total trauma severity × neuroticism	.11	.001	

Note. ^a^0 = female, 1 = male.

**Fig 1:**
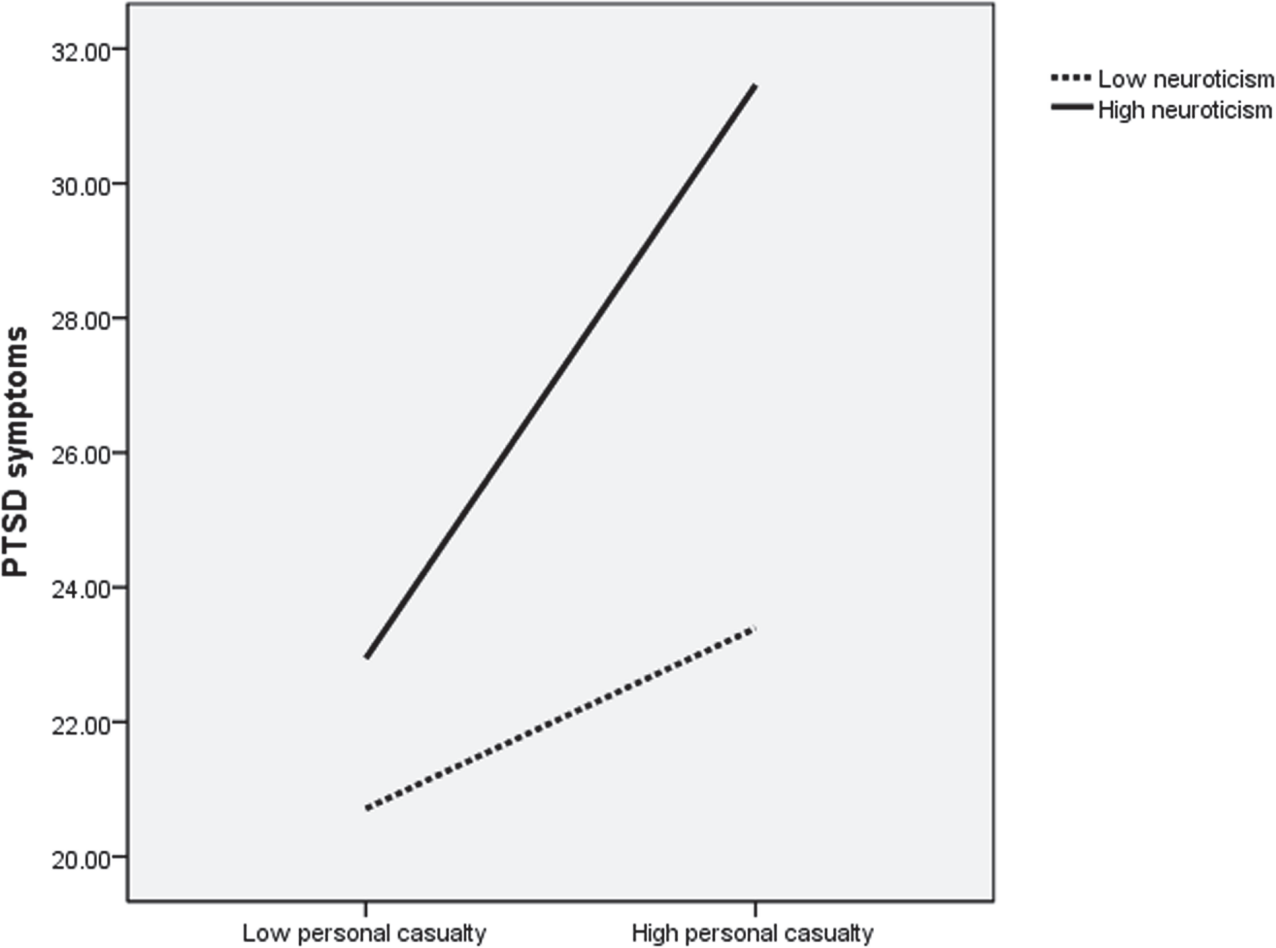
PTSD symptoms: Personal casualty×Trait neuroticism. This figure presents a two-way ANOVA investigating the association of posttraumatic stress disorder (PTSD) symptoms with the interaction between neuroticism scores and the presence of personal casualty. Effect sizes: neuroticism (*F* = 21.33; *P* <. 001; partial *η* ^2^ = 0.031), personal casualty (*F* = 25.20; *P* <. 001; partial *η* ^2^ = 0.037), neuroticism by personal casualty (*F* = 6.84; *P* = .009; partial *η* ^2^ = 0.010). Given similar levels of personal casualty, high neuroticism adolescents had higher PCL-C scores compared to those with low neuroticism. Moreover, the separation of PCL-C scores appeared more pronounced with increasing levels of severity of personal casualty.

**Fig 2:**
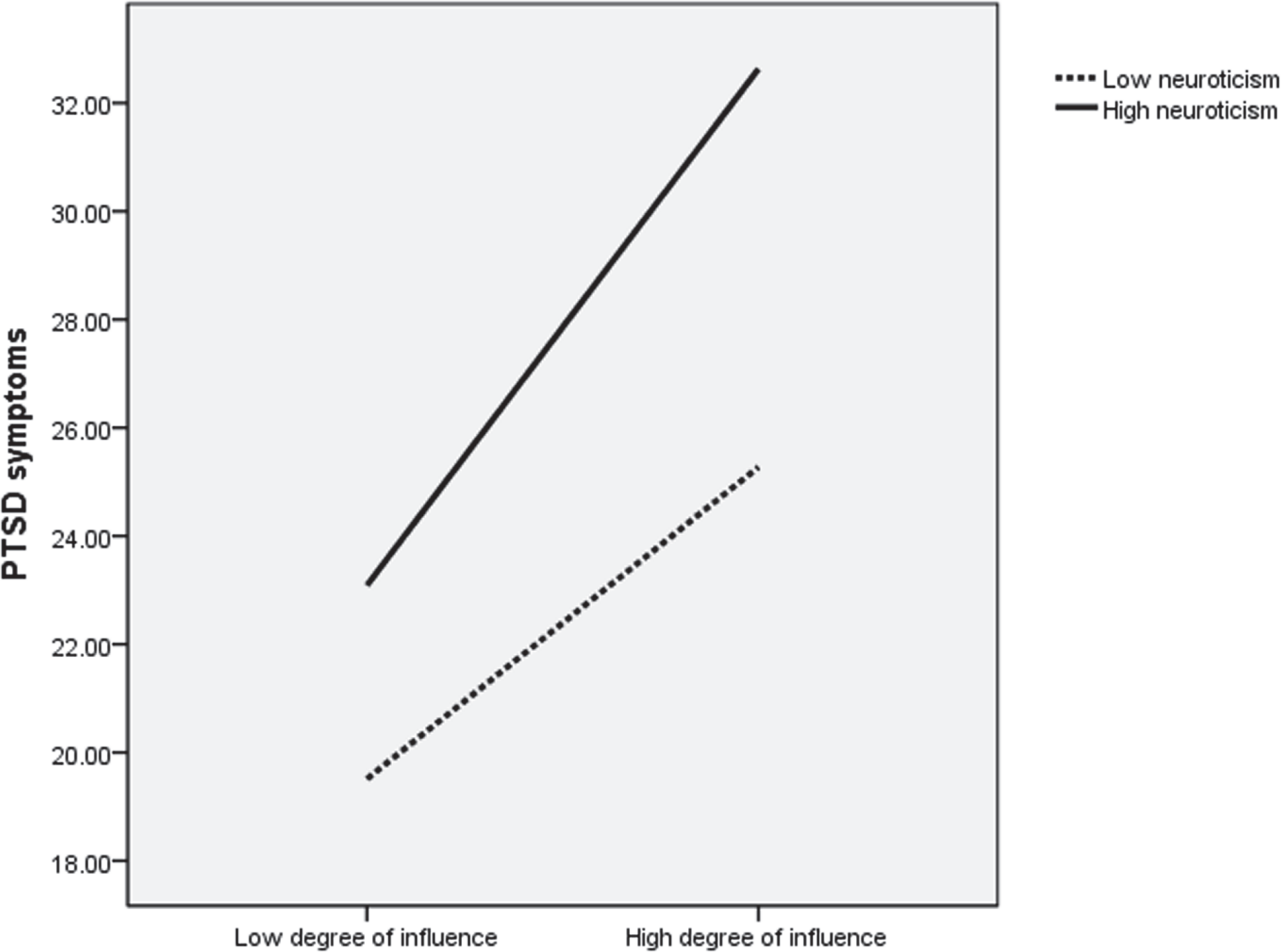
PTSD symptoms: Degree of influence×Trait neuroticism. This figure presents a two-way ANOVA investigating the association of posttraumatic stress disorder (PTSD) symptoms with the interaction between neuroticism scores and degree of influence. Effect sizes: neuroticism (*F* = 37.40; *P* <. 001; partial *η* ^2^ = 0.054), degree of influence (*F* = 73.35; *P* <. 001; partial *η* ^2^ = 0.101), neuroticism by degree of influence (*F* = 4.49; *P* = .035; partial *η* ^2^ = 0.007). Given a similar degree of influence, high neuroticism adolescents had higher PCL-C scores compared to those with low neuroticism. Moreover, the separation of PCL-C scores appeared more pronounced with increasing degree of influence.

**Fig 3:**
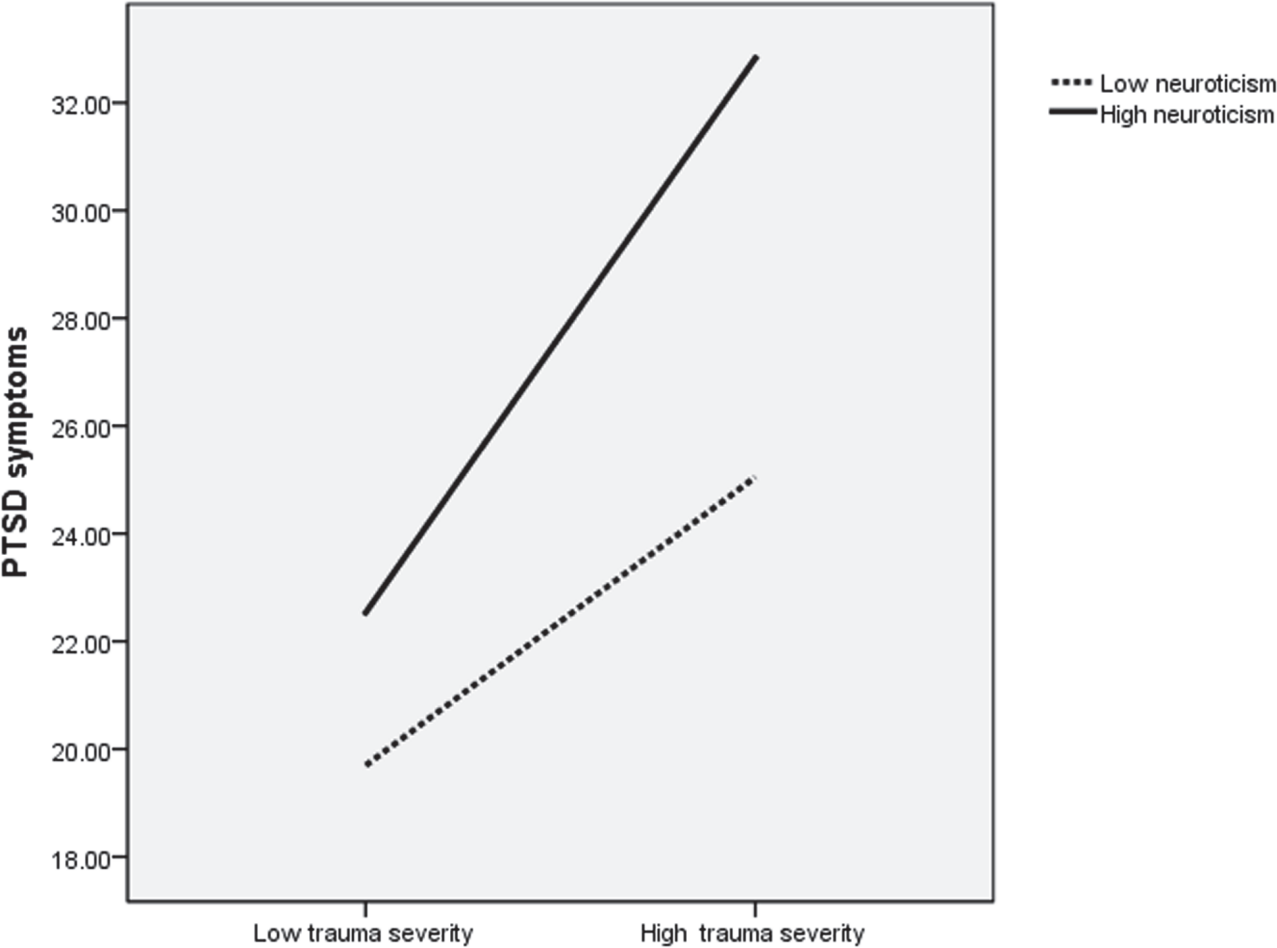
PTSD symptoms: Trauma severity×Trait neuroticism. This figure presents a two-way ANOVA investigating the association of posttraumatic stress disorder (PTSD) symptoms with the interaction between neuroticism scores and trauma severity. Effect sizes: neuroticism (F = 35.49; P <. 001; partial η2 = 0.051), trauma severity (F = 77.13; P <. 001; partial η2 = 0.105), neuroticism by trauma severity (F = 7.70; P = .006; partial η2 = 0.012). Given similar levels of trauma severity, high neuroticism adolescents had higher PCL-C scores compared to those with low neuroticism. Moreover, the separation of PCL-C scores appeared more pronounced with increasing trauma severity.

For better visual conceptualization of the significant interactions, the three indicators of trauma severity and trait neuroticism were dichotomized as high or low using median values. A series of two-way ANOVAs were then performed, including high or low trait neuroticism and one of the three indicators of trauma severity. All main effects and interactions were statistically significant (*P* <. 05). Figs. 1–3 show that given similar levels of trauma exposure, high neuroticism adolescents had higher PCL-C scores compared to those with low neuroticism. Moreover, the separation of PCL-C scores appeared more pronounced with increasing levels of severity of trauma exposure.

## Discussion

In this cross-sectional school-based study of 662 adolescents exposed to an accidental explosion, we assessed the prevalence of PTSD symptoms, and more specifically, the involvement of trauma severity and trait neuroticism (and their interrelationship) in the development of PTSD symptoms (controlling for gender and age), using hierarchical regression models. We hypothesized that the interaction between these factors might be associated with PTSD symptoms. The results confirmed our hypotheses, as they indicate that the interaction between trait neuroticism and trauma severity is related to PTSD symptoms, and the association between trauma severity and PTSD symptoms is most pronounced in individuals with high levels of neuroticism. Similar findings were found when we dichotomized trait neuroticism and trauma severity and further explored the main effect and interactions.

In the sample of trauma-exposed adolescents, the prevalence of PTSD symptoms was around 13%, which is relatively lower than that found in earlier studies [28,54]. Specific factors, such as individual characteristics, the nature of the accident, exposure severity, etc., may account for this discrepancy. The majority of our participants were only witnesses to the accident; thus, it is possible that they experienced less severe trauma. Many studies have found that girls exposed to traumatic events are more likely to develop PTSD than are boys [6,55]; our results provide support for this earlier research. Scholars have hypothesized that the greater vulnerability of girls to developing PTSD may be attributed to gender differences in coping styles [56] or their propensity to exhibit higher levels of neuroticism and anxiety [57]. Additionally, findings of the present study converge with previous research regarding victims of the Wenchuan earthquake, which revealed that individuals aged 13 to 18 years had higher PCL-C scores than those under age 13.

Our results are consistent with findings of prior studies [58–61], which indicated that after controlling the effect of age and gender, all aspects of trauma exposure were significantly positively related to subsequent PTSD symptoms. These findings provide empirical support for the ‘‘dose-response effect” [62]. In other words, higher levels of trauma exposure usually contribute to greater PTSD symptoms. It has been postulated that at higher levels of exposure to earthquakes, adolescents would re-experience horrible memories of catastrophe, injury, and death later in their lives and would be more likely to develop PTSD symptoms [15,42,63].

Regarding personality features, we observed a strong association between level of neuroticism and risk of PTSD symptoms, which corroborates the previous findings of Cox et al. (2004) [64] and Perrin et al. (2014) [65]. Results from two prospective investigations confirmed the role of neuroticism in the development of PTSD following traumatic experiences [47,66]. High neuroticism individuals appear to be more reactive to adverse events [67] and tend to be very ruminative, especially regarding their health and well-being [68], thus leading them to experience more distress across time and regardless of the situation [69]. Moreover, given their inclinations towards emotion-focused coping, individuals high in neuroticism engage in fewer health practices relative to those who are less neurotic [70].

Specifically, we found that the interaction between high trait neuroticism and severe trauma exposure was associated with high PTSD symptom severity. That is, the association between trauma severity and PTSD symptoms is most pronounced in individuals with high levels of neuroticism. This finding is similar to results of previous studies regarding psychopathology. For example, Ormel et al. (2001) found that in the presence of high neuroticism, the depressogenic effect of stressful life events was substantial [48]. Brietzke et al. (2012) concluded that early interpersonal trauma interacts with genetic predisposition in affecting the development and expression of schizophrenia [71].

Our research should be interpreted in light of several limitations. First, in assessing PTSD symptoms, we applied the PCL-C, which is a screening tool rather than a standard clinical diagnostic method; thus, incidence of PTSD was probably somewhat overestimated. Second, retrospective assessment implies potential recall bias regarding exposure to traumatic events and the presence of PTSD symptoms. Also, the assessment of trait neuroticism could have been affected by the occurrence of PTSD symptoms. Future studies are therefore needed to highlight the role of clinical diagnostic measurements when addressing similar issues, to enhance the reliability of the findings among clinicians.

Despite these possible limitations, the current study also has several strengths. Above all, our study is among the first investigations, to our knowledge, to report the prevalence of PTSD symptoms in a relatively large sample of adolescent survivors after an accidental explosion; the large sample size lends reliability to the results. Furthermore, we focused on interactions between trauma severity and trait neuroticism in predicting PTSD symptoms following an accidental explosion; these results may provide a better understanding of the origins of adolescent mental health and illness.

## Conclusions

In conclusion, our results suggest that both trauma severity and trait neuroticism influence subsequent PTSD symptoms after trauma exposure, and that trait neuroticism seems to moderate the association between trauma severity and PTSD symptoms. Therefore, findings of the current research may have important clinical implications for preventing and treating stress-related disorders in adolescents in the aftermath of traumatic accidents. Mental health professionals should offer support to reduce the development of PTSD among adolescents after accidental explosions, especially for those who witnessed injury or death, or lost family members, houses, or property during the incident. Education and interventions could also be employed to help adolescent survivors cope with PTSD.

## Supporting Information

S1 Dataset (XLS)
